# Mapping of quantitative trait locus for resistance to anthracnose in a population derived from genotypes PI 527538 and Ervilha of common bean

**DOI:** 10.3389/fpls.2025.1691703

**Published:** 2025-12-10

**Authors:** Mwiinga Mulube, Swivia Hamabwe, Kuwabo Kuwabo, Modreen Chinji, Mukuni Nkandela, Joseph Botha, Brian Mwense, Langa Tembo, Davies Lungu, Chikoti Mukuma, Travis Parker, Kelvin Kamfwa

**Affiliations:** 1 Department of Plant Sciences, University of Zambia, Lusaka, Zambia; 2 Zambia Agricultural Research Institute, Kasama, Zambia; 3 Department of Plant Sciences, University of California, Davis, Davis, CA, United States

**Keywords:** *Colletotrichum lindemuthianum*, quantitative trait locus, candidate genes, recombinant inbred lines, race

## Abstract

Anthracnose (ANT) caused by the fungus *Colletotrichum lindemuthianum* is a major disease of common bean (*Phaseolus vulgaris* L.). The objectives of this study were to (i) characterize four isolates of *C. lindemuthianum* into races, (ii) identify quantitative trait locus (QTL) associated with resistance to these four races, and (iii) identify lines with pyramided resistance to the characterized races. The four isolates collected from the major bean-growing region of Zambia were characterized into races 6, 7, 81, and 294. This is the first time that these four races are being reported in Zambia. These races were used to inoculate 220 F_5:9_ recombinant inbred lines (RILs) derived from a cross between the Andean common bean genotypes PI 527538 and Ervilha. Only two RILs were highly resistant to all four races. Six QTLs were identified on chromosomes Pv01 (ANT1.1), Pv03 (ANT3.1), Pv04 (ANT4.1), and Pv10 (ANT10.1, ANT10.2, and ANT10.3), which conferred resistance to the four characterized races. The *R*^2^ of these QTLs ranged from 6.3% to 90.3%, suggesting that both major- and minor-effect loci controlled ANT resistance. Some of the identified QTLs overlapped with previously reported QTLs while others did not. A total of 31 disease resistance genes with NB-ARC-LRR and TIR-NBS-LRR domains were identified as candidate genes for ANT1.1 and ANT10.2. The two RILs with superior resistance to all four races represent a valuable genetic resource to improve the yellow beans for ANT resistance while QTL analysis has provided valuable information to develop a marker-assisted selection strategy for ANT resistance in the yellow bean market class.

## Introduction

Common bean (*Phaseolus vulgaris* L.) is an important crop in many countries (Uebersax et al., 2022). It is a major source of protein, fiber, carbohydrates, and micronutrients (iron and zinc) (Broughton et al., 2003; Buruchara et al., 2011). Common bean is genetically diverse and is classified into several market classes mainly based on seed characteristics. Yellow beans are a major market class of common bean particularly in Africa (Sichilima et al., 2016). Yellow beans have become popular with consumers due to their faster cooking time and nutritional superiority to other market classes (Sichilima et al., 2016; Wiesinger et al., 2018). They contain high levels of iron, a nutrient that is deficient in most African diets, but most of this iron has high bioavailability (Wiesinger et al., 2016; Sadohara et al., 2022). Despite their nutritional superiority, the productivity of yellow beans remains low.

Anthracnose (ANT) caused by the fungus *Colletotrichum lindemuthianum* is a major contributor to this low productivity of the yellow beans. It can cause yield losses of up to 100% in highly susceptible genotypes and when environmental conditions favor its spread (Singh and Schwartz, 2010). ANT is a seedborne disease that can be controlled effectively with the use of fungicides (Mohammed, 2013). However, chemical control is not affordable for the majority of smallholder farmers. Development and use of resistant varieties is the most cost-effective control strategy for ANT (Singh and Schwartz, 2010). Although ANT resistance is present in many other market classes, transferring this resistance into the yellow bean background remains challenging due to the poor recovery of the yellow seed coat (particularly the Manteca type) when yellow beans are crossed with other market classes. Therefore, identifying sources of resistance within the yellow market class and elucidating the genetic basis of that resistance could play a critical role in improving ANT resistance in yellow beans.

Extensive variability in *C. lindemuthianum* exists at several levels including within field, across production regions within a country, and at both regional and global scales. A recent study aimed at understanding the race structure of *C. lindemuthianum* in Zambia reported 50 races within the country (Sansala et al., 2024). Globally, more than 300 races have been reported (Nunes et al., 2021; Sansala et al., 2024). Previous studies on race characterization have revealed an intra-race diversity, suggesting that virulence diversity is greater than what is captured by classifications based solely on differential cultivars (Sansala et al., 2024; Mahuku and Riascos, 2004). These races are generally classified as either Andean or Middle American based on the gene pool of the host genotype from which they were isolated. Typically, Andean races are isolated from Andean genotypes and are highly virulent on Andeans. Middle American races are isolated from Middle American genotypes and highly virulent on Middle American genotypes though they also attack Andean genotypes and tend to exhibit higher virulence diversity than Andean races. Studies aimed at understanding the genetic variability of *C. lindemuthianum* in specific production environments as well as identifying sources of resistance are critical for the development of varieties with durable ANT resistance.

Resistance to ANT is controlled by mainly major-effect resistance loci named with the prefix “*Co*”. To date, 23 major-effect loci have been identified across eight chromosomes (Ferreira et al., 2013; Badiyal et al., 2024). Resistance conferred by these genes follows a gene-for-gene model and is typically associated with clusters of resistance genes containing NBS-LRR domains. These clusters provide race-specific qualitative resistance (Geffroy et al., 1999; Rodríguez-Suárez et al., 2008; Mungalu et al., 2020; Ferreira et al., 2023; Kuwabo et al., 2023; Sinkala et al., 2024). The *Co* genes have been classified as either Andean or Middle American (Young and Kelly, 1996). In addition to these major-effect loci, minor-effect quantitative trait loci (QTLs) have also been identified. Both major- and minor-effect resistance loci provide resistance to specific races of *C. lindemuthianum*, and there is no single locus that is effective against all races.

One effective strategy for developing bean varieties with durable resistance for a specific region is to first characterize the local race structure of *C. lindemuthianum* and then conduct genetic studies such as genome-wide association studies (GWAS) or biparental population QTL analyses to identify resistance loci and/or genes for the identified races. This information can then be used to develop a gene pyramid that can confer effective and durable ANT resistance. QTL mapping has widely been used to identify genomic regions conferring ANT resistance. Some of the identified QTL have co-localized with known *Co* major ANT resistance loci or previously identified QTL while others are novel (Mungalu et al., 2020; Kachapulula et al., 2025). The QTL analyses results involving multiple races have provided better insights into specific genomic regions that provide resistance to specific races of *C. lindemuthianum* (Mungalu et al., 2020; Kachapulula et al., 2025).

The yellow Andean genotypes PI 527538 and Ervilha show significant variation in their responses to different races of *C. lindemuthianum* under both artificial and natural infection conditions. PI 527538 has a Njano yellow seed coat color while Ervilha has a pale yellow (Manteca) seed coat color, which is strongly preferred by consumers in Africa (Sichilima et al., 2016). The genetic basis of the observed variation in ANT resistance between PI 527538 and Ervilha, and their recombinant inbred lines (RILs) remains unknown. The objectives of this study were to (i) characterize four isolates of *C. lindemuthianum* into races, (ii) identify QTLs associated with resistance to these four races, and (iii) identify lines with pyramided resistance to the characterized races.

## Materials and methods

### Plant materials

Twelve differential cultivars, four Andean and eight Middle American, were used for race characterization as described by Pastor-Corrales (1991). **Table 1** presents the list of these 12 races differential cultivars, their gene pool, resistance genes, binary numbers, and numeric value used to assign race numbers based on their susceptibility to a specific isolate (Young and Kelly, 1996).

**Table 1:** Reaction of the 12 race differentials to inoculation with *Colletotrichum lindemuthianum* races 6, 7, 81, and 294.

Race differential	Gene pool	Gene	Binary number	Race/Isolate name			
				6	7	81	294
				A23-2	A23-1	22-4-B	A23-16
Michelite	MA	Co-11	1	1	9	9	1
MDRK	A	Co-1	2	8	9	1	9
Perry Marrow	A	Co-1^3^	4	8	9	1	9
Cornell 49242	MA	Co-2	8	1	1	1	1
Widusa	A	Co-1^5^	16	1	1	9	1
Kaboon	A	Co-1^2^	32	1	1	1	9
Mexico 222	MA	Co-3	64	1	1	9	1
PI 20262	MA	Co-3^3^; Co-4^3^	128	1	1	1	1
TO	MA	Co-4	256	1	1	1	9
TU	MA	Co-5	512	1	1	1	1
AB 136	MA	Co-6; Co-8	1,024	1	1	1	1
G2333	MA	Co-3^5^; Co-4^2^, Co-5^2^	2,048	1	1	1	1

MA, Middle American gene pool; A, Andean gene pool.

This study used a total of 220 F_5:9_ RILs derived from a cross between the Andean genotypes PI 527538 and Ervilha to identify QTL conferring resistance to C. *lindemuthianum* races. However, the number of RILs evaluated varied from 132 (race 81) to 220 (race 6). This variation was caused by differences in germination and also some genotypes not reaching the scoring stage. This population was developed using Single Seed Descent method at Michigan State University (Bassett et al., 2021). PI 527538 was originally collected from Burundi and has a large seed size (48 g/100 seeds) with a green-yellow color and type I (bush) growth habit (Cichy et al., 2015). Ervilha is an Angolan landrace, with a large seed size (58.2 g/100 seeds) Manteca (pale yellow) seed color and a type I growth habit.

### Isolation, race characterization, and reaction of the RILs to characterized races

Pods that showed typical ANT symptoms such as black lesions with slightly sunken center and dark brown or purplish brown margins (Mohammed, 2013) were collected from a bean field at Malashi Research Station (latitude = −11.80; longitude = 31.45) in the Mpika district of Muchinga province of Zambia, a major bean-growing region. The bean field from where the samples were collected is located at Malashi Research Station, which is a major testing site for disease resistance of the breeding lines from public breeding programs in Zambia. The pods were collected from different Andean breeding lines belonging to the University of Zambia (UNZA) Common Bean Breeding program grown in two consecutive growing seasons. Infected pods were transported to the bean laboratory at UNZA, where fungal isolations were conducted. A previously described protocol (Sansala et al., 2024; Nalupya et al., 2021; Mungalu et al., 2020) was used for fungal isolation and race characterization. Small pieces from the lesion (0.5 to 2 mm) were cut out. These were surface-sterilized (for 2 min) using 1% sodium hypochlorite and 70% ethanol, and then rinsed in distilled water.

The pieces were then dried at room temperature on paper towel for 30–60 min and incubated on Petri dishes containing potato dextrose agar (PDA) media (39 g/L) in the dark for 10 days to allow sporulation. Purified isolates were obtained by streaking diluted spore suspensions from the sporulated plates onto fresh plates. After 24 to 48 h, a single germinating spore was removed from each plate and transferred to a new plate containing fresh PDA to establish single-spore cultures. The purified cultures were kept in a dark cupboard for 10 days until *C. lindemuthianum* had sporulated. Distilled water was added to the plates and then the spores were dislodged by carefully scraping the top of the mycelia using a glass rod. The scraped-off spore suspension water was filtered using a cheese cloth. The filtered spore suspension concentration was measured using a hemacytometer and adjusted to a concentration of 1.2 × 10^6^ spores per mL. Tween 20 (0.01%) was then added to the spore suspension (10 μL per 100 mL), which was then ready for simultaneous inoculation of the race differentials, checks, parents, and RILs.

The race differentials, RILs, parents, and checks were planted on Styrofoam trays that had 60 wells (5 cm wide, 5 cm long, and 5 cm deep) filled with sterilized soil from the field. Three genotypes (Kabulangeti, Lusaka, and G2333) were used as checks. Kabulangeti and Lusaka are Andean Zambian landraces and highly susceptible to ANT. Kabulangeti is purple speckled with a type III growth habit while Lusaka is a Manteca yellow variety with a type I growth habit. G2333 is Middle American with a type III growth habit and is highly resistant to ANT (Young et al., 1998).

A completely randomized design with three replications was used. Each replication was composed of two seeds (two seedlings) of each genotype planted in a well. Therefore, a total of six seedlings per genotype were evaluated across the three replications. The seedlings were inoculated when their dicotyledonous leaves had expanded fully. A handheld sprayer filled with inoculum was used to spray the seedling canopy, ensuring that the underside of the seedlings’ leaves was also sprayed. After the spray, seedlings were kept at room temperature for 2 h to allow inoculum to dry before they were transferred to the high humidity chamber (95%–100% humidity) where they were kept for 72 h. From the humidity chamber, the trays were transferred to the greenhouse, which was maintained at ambient temperature and humidity with approximately12 h of natural light to allow for disease development. ANT severity on the seedlings was then scored on a CIAT scale of 1–9 (van Schoohoven and Pastor-Corrales, 1987). A score of 1 was for seedlings with no visible symptoms, a score of 2–3 was for seedlings showing limited necrotic, a score of 4–8 was for seedlings with small to large sporulating lesions, and a score of 9 was for severely infected and dead seedlings. Scores of 1–3 were considered highly resistant; 4–6, moderately resistant; and 7–9, highly susceptible. For race characterization, race differentials with a severity score of 1–3 were considered resistant while a score of 4–9 was considered susceptible (Sansala et al., 2024).

### Severity score analysis

Analysis of variance (ANOVA) was conducted in R (R Core Team, 2025) software on the severity scores to determine the statistical significance of their differences in their response to the four races. Because the data were not normally distributed, they were first transformed using logarithmic transformation before they were used in ANOVA.

Pearson correlation analysis for severity scores among the four races was conducted using a base package in R software to determine relatedness.

Broad-sense heritability for each trait was calculated using the variance components, which were computed using the R package lme4 and the following equation:


$$H^{2}=\frac{\sigma_{g}^{2}}{\sigma_{g}^{2}+\sigma_{e}^{2}/r}$$

where *H*^2^ is broad-sense heritability, $\sigma_{g}^{2}$ was the variance component for genotype, $\sigma_{e}^{2}$ was the error variance component, and *r* is the number of replications.

### Genotyping and QTL analysis

The population used in this study was previously genotyped and details on how this was conducted can be found in Bassett et al. (2021). Briefly, DNA was harvested from fresh leaves using a DNA extraction kit Macherey-Nagel NucleoSpin Plant II. The DNA was genotyped using the BARCBean12K BeadChip with 12,000 SNPs at the USDA Beltsville Agricultural Research Center in Beltsville, MD, USA. A total of 870 SNPs were polymorphic and were used to build 11 linkage groups using the software MapDisto version 2.17 (Lorieux, 2012).

The size of the linkage map ranged from 25.8 cM (Pv10) to 52.8 cM (Pv02), with a total distance of 439.3 cM for 11 linkage groups. The number of SNPs per linkage group ranged from 24 (Pv05 and Pv11) to 138 (Pv03) (**Supplementary Table S1**). Composite Interval Mapping in the software R/qtl (Broman et al., 2003) was used to map the QTL for resistance to the four characterized races 6, 7, 81, and 294. To determine the threshold for QTL significance, 1,000 permutations were conducted. A logarithm of odds (LOD) score of 3 was used. The software Mapchart was used to plot the linkage maps with QTL on them (Voorrips, 2002).

### Candidate gene identification

Candidate genes were identified using a previously described method (Kuwabo et al., 2023). JBrowse in Phytozome was used to browse the *P. vulgaris* v2.1 genome to identify positional candidate genes. A gene was considered as a candidate if it was located within this QTL region (±250 kb of the QTL peak) and its product is predicted to function in disease resistance.

## Results

### Race characterization

The four isolates A23-2, A23-1, A23-16, and 22-4-B that were infected on the race differentials were characterized as races 6, 7, 81, and 294, respectively (**Table 1**). Race 6 is an Andean race that was virulent on Andean differentials MDRK and Perry Marrow. The races 7, 81, and 294 were classified as mixed races because they were virulent on both Andean and Middle American differentials as previously described by Zuiderveen et al. (2016) and Sansala et al. (2024) (**Table 1**).

### Response of the RILs, parents, and checks to inoculation with races 6, 7, 81, and 294

**Figure 1:**
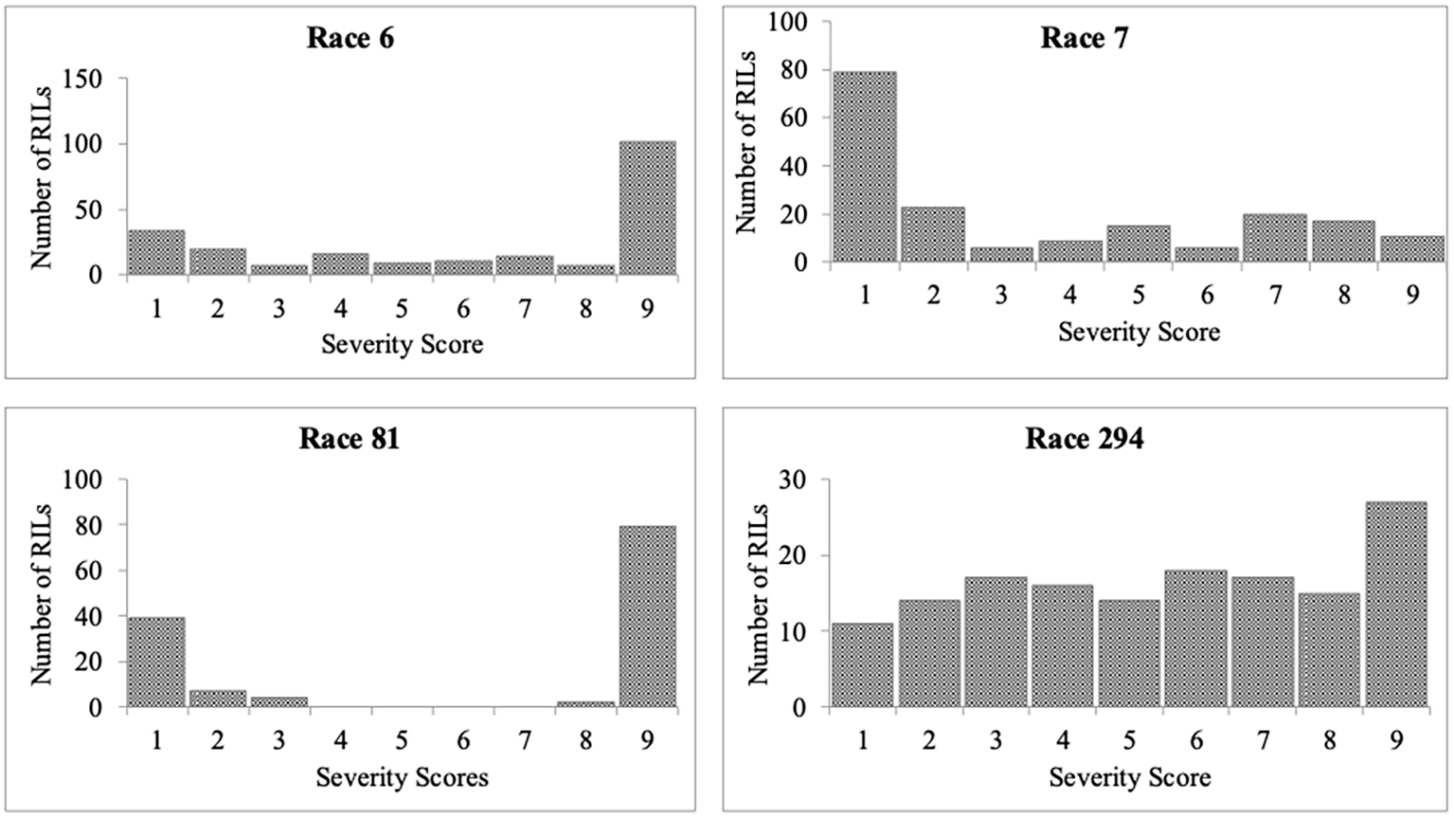
Frequency distributions of severity scores for *Colletotrichum lindemuthianum* races 6, 7, 81, and 294 inoculated on recombinant inbred lines derived from a cross of PI 527538 and Ervilha. This should be the bare graph (also known as histogram).

Significant differences were observed among the RILs in their reaction to the four races. The average severity score for the population for race 6 was 5.9 (**Table 2**). Among the 220 RILs that were evaluated, 61 were highly resistant, 36 were moderately resistant, and 123 were susceptible (**Figure 1**). The parent PI 527538 was highly resistant (score of 2.3) while Ervilha was highly susceptible (score of 9) to race 6 (**Table 1**). The susceptible checks Kabulangeti and Lusaka had a score of 9 while G2333 had a score of 1. Reaction of RILs to race 6 followed a bimodal distribution (**Figure 1**).

**Table 2:** Mean and range for anthracnose severity measured on parents (PI 527538 and Ervilha), checks (G2333, Kabulangeti, and Lusaka) and recombinant inbred lines inoculated with *Colletotrichum lindemuthianum* races 6, 7, 81, and 294.

	Parents			Checks			RILs (*n* = 132–220)		
Race/Isolate	PI 527538	Ervilha	*t*-test	G2333	Kab	Lusaka	Mean	Range	ANOVA
Race 6	2.3	9.0	***	1	9	9	5.9 ± 0.1	1.0–9.0	***
Race 7	7.0	4.2	*	1	9	9	3.5 ± 0.1	1.0–9.0	***
Race 81	1.0	7.5	***	1	9	9	6.2 ± 0.2	1.0–9.0	***
Race 294	2.0	5.0	***	1	9	9	5.2 ± 0.1	1.0–9.0	***

Severity scores were on a scale of 1–9; G2333 = resistant check; Kab (Kabulangeti) = susceptible check; Lusaka = susceptible check; ANOVA = analysis of variance; * = 0.05 (level of significance); ** = 0.01 (level of significance) and *** = 0.001 (level of significance); *n* = number; RILs, recombinant inbred lines.

For race 7, severity scores ranged from 1 to 9 with a population mean of 3.5 (slightly skewed to resistance). Among the 186 RILs that were inoculated with race 7, 108 were highly resistant (severity score of 1–3), 30 were moderately resistant (severity scores of 4–6), and 48 were highly susceptible (scores of 7–9). Ervilha had a severity score of 4.2 (moderately resistant) while PI 527538 was susceptible with a score of 7. The checks reacted as expected, as the susceptible checks Kabulangeti and Lusaka were highly susceptible (score of 9) while the resistant check G2333 was highly resistant (score of 1).

The severity score for race 81 ranged from 1 to 9 with a population mean of 6.2. Of the 132 RILs that were evaluated for response to this race, 50 were highly resistant while 82 were highly susceptible, with nothing in the moderate category, thereby showing a bimodal distribution pattern of the severity scores (**Figure 1**). The parent PI 527538 was highly resistant (severity score of 1) while Ervilha was susceptible (severity score 7.5), and the *t*-test showed that these two means were significantly different. The checks reacted as expected with the two susceptible checks having a score of 9 while the resistant G2333 had a score of 1.

The mean severity score for the population inoculated with race 294 was 5.2. Of the 149 RILs evaluated for their response to this race, 42 were highly resistant, 48 were moderately resistant, and 59 were highly susceptible. The parent PI 527538 was highly resistant (score of 2) while Ervilha was moderately resistant (score of 5).

Transgressive segregation was observed for all four races on both ends of the severity score spectrum. The RILs YY_49 and YY_65 were the only two that were highly resistant to all four races as indicated in **Supplementary Table S2**.

The highest correlation in severity score was observed between races 6 and 7 (*r* = 0.41) while the lowest correlation was between races 6 and 294 (*r* = 0.08) (**Table 3**).

**Table 3:** Pearson correlations of severity scores for *Colletotrichum lindemuthianum* races 6, 7, 81, and 294 inoculated on recombinant inbred lines derived from a cross of PI 527538 and Ervilha.

Race id	Race 6	Race 7	Race 81	Race 294
Race 6	1	0.41	0.29	0.08
Race 7	–	1	0.25	0.13
Race 81	–	–	1	−0.18
Race 294	–	–	–	1

Broad-sense heritability ranged from 0.90 (race 6) to 0.96 (race 81) with an average of 0.93.

### QTL analyses

**Figure 2:**
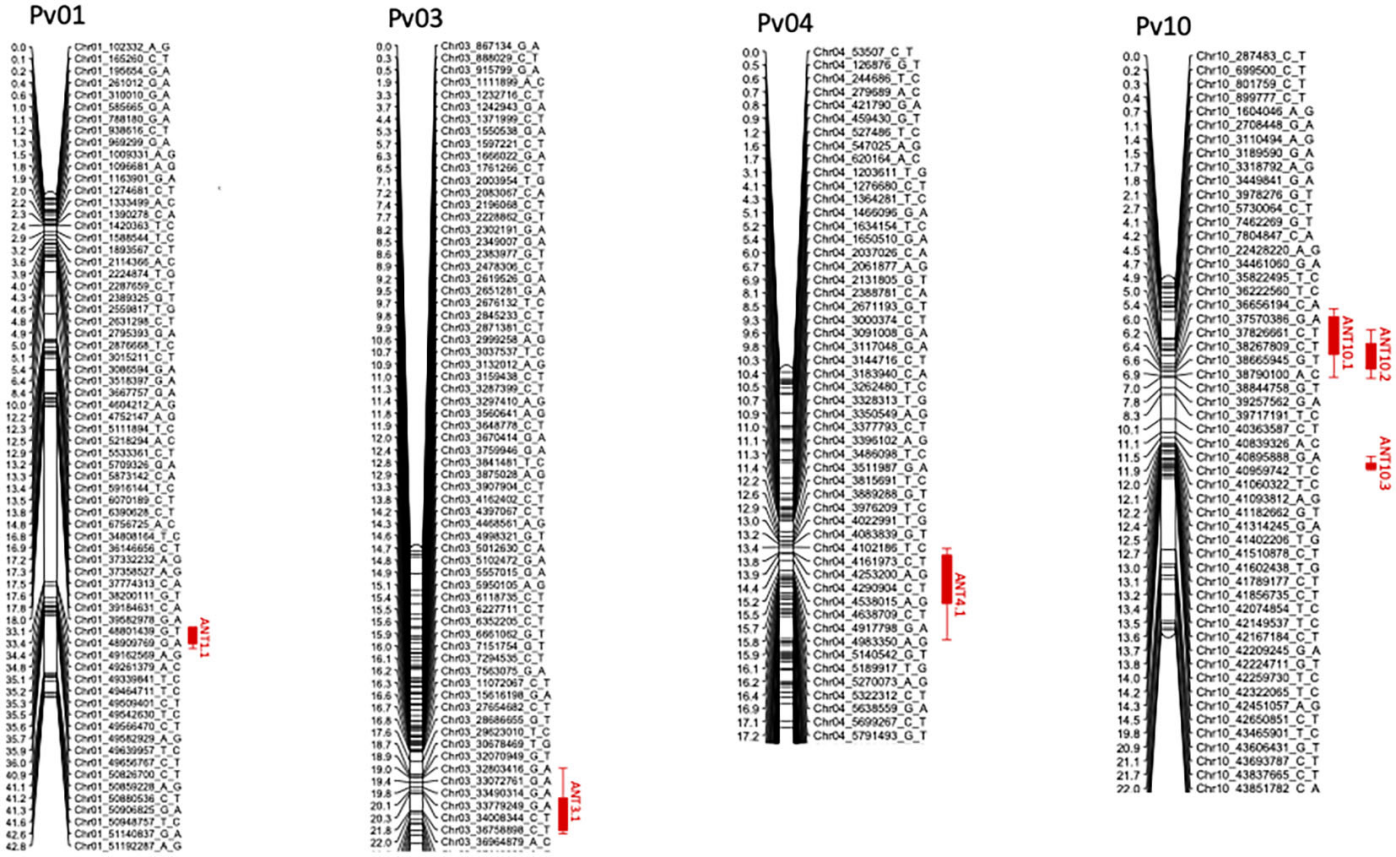
Linkage maps for chromosomes Pv01, Pv03, Pv04, and Pv10 with positions for QTL ANT1.1 (for races 6 and 81), ANT3.1 (race 294), ANT4.1 (race 6), ANT10.1 (race 6), ANT10.2 (race 7), and ANT10.3 (race 294). This should for the linkage maps.

A total of six QTLs on chromosomes Pv01, Pv03, Pv04, and Pv10 were identified as having provided resistance to races 6, 7, 81, and 294 (**Table 4**; **Figure 2**).

**Table 4:** Quantitative trait loci for resistance to *Colletotrichum lindemuthianum* races 6, 7, 81, and 294 identified in a population of recombinant inbred lines derived from a cross of PI 527538 and Ervilha.

QTL name	CHR	Race/Isolate	Peak SNP	QTL peak cM (Mbp)	QTL interval (Mbp)	LOD	*R*^2^ (%)
ANT1.1	Pv01	6	Chr1_49509401	35.3(49.5)	48.8–50.8	6.9	11.4
ANT1.1	Pv01	81	Chr1_49656767	36.0 (49.8)	48.8–50.8	92.8	90.3
ANT3.1	Pv03	294	Chr3_3670414	12.0 (36.7)	34.0–37.4	3.3	6.9
ANT4.1	Pv04	6	Chr4_527486	1.2 (5.3)	4.3–7.6	3.9	6.4
ANT10.1	Pv10	6	Chr10_22428220	4.5 (22.4)	5.3–39.5	4.1	6.3
ANT10.2	Pv10	7	Chr10_699500	0.1 (3.8)	3.7–3.9	6.1	12.2
ANT10.3	Pv10	294	Chr10_42209245	13.7 (42.2)	40.6–43.5	12.8	29.3

QTL , quantitative trait locus; Mbp, million base pairs; LOD, logarithm of odds; CHR, chromosome; *R*^2^, proportion of phenotypic variance explained by the QTL; %, percentage.

A major QTL (ANT1.1) was identified at the end on Pv01 (48.8-50.8 Mbp). ANT1.1 provided resistance to both races 6 and 81. The LOD score (statistic metric for the presence or absence of QTL) for ANT1.1 was 6.9 and 92.8 for races 6 and 81, respectively. This was a major QTL that explained 11.4% and 90.3% of resistance in the population to races 6 and race 81, respectively. The resistance allele came from the parent PI527538. The major effect of ANT1.1 depicted in the large *R*^2^ value is consistent with the observed bimodal distribution of the severity scores, which suggested resistance provided by a major effect gene. A total of 17 genes with the NB-ARC-LRR domain belonging to a family of disease resistance genes (**Supplementary Table S3**) were identified within the confidence of ANT1.1.

A minor-effect QTL ANT3.1 was identified on Pv03 (34.0-37.4 Mbp). This QTL explained 6.9% of the variation in resistance to race 294. The parent PI 527538 provided the resistance allele at this QTL.

The QTL ANT4.1 was identified near the beginning of Pv04 (4.3-7.6 Mbp). This QTL explained approximately 6.4% of the variation in resistance to race 6 among the RILs. The parent PI 527538 provided resistance at this QTL.

There were three QTLs (ANT10.1, ANT10.2, and ANT10.3) that were identified on Pv10. ANT10.1 was located at the beginning of the chromosome (3.7–3.9 Mbp) and was a minor QTL that explained approximately 6.3% of the variation in severity score for race 6 among the RILs. ANT10.2, which provided resistance to race 7, was also located at the beginning of the chromosome, and it was also a major QTL with an *R*^2^ of 12.2%. The third QTL to be identified on Pv10 was ANT10.3, and was a major QTL that explained 29.3% of the resistance among the RILs to race 294. The parent Ervilha contributed the resistant allele at ANT10.1 while PI527538 contributed the allele at ANT10.2 and ANT10.3. A total of 14 resistance genes with NB-ARC-LRR and TIR-NBS-LRR domains (**Supplementary Table S3**) were identified within the confidence of ANT10.2.

## Discussion

In this study, four isolates from Zambia were characterized into four diverse races (races 6, 7, 81, and 294). Though nine previous studies (Zulu, 2005; Kachapulula, 2010; Mungalu et al., 2020; Nalupya et al., 2021; Sansala et al., 2024; Mwense et al., 2024; Kuwabo et al., 2023; Sinkala et al., 2024; Kachapulula et al., 2025) have reported the occurrence of a diverse a number of *C. lindemuthianum* races in Zambia, this is the first report of the existence of the four races 6, 7, 81, and 249 in Zambia. Race 6 was an Andean race that was virulent on only Andean differentials while the other three races (7, 81, and 294) were mixed as they were virulent on both Andean and Middle American differentials. The characterization of Andean races was consistent with the widespread growing of Andean beans in Zambia. The four characterized races are diverse despite coming from a small geographic area and in two growing seasons, which demonstrated how highly variable *C. lindemuthianum* can be even in a small geographic area. This high virulence diversity poses a challenge to the development of varieties with durable resistance. All four identified races in the current study have previously been reported in other countries outside Zambia including the United States (Zuiderveen et al., 2016) and Brazil (Paulino et al., 2022). Race 81 has previously been reported to be highly frequent in Brazil (Paulino et al., 2022).

There were several genotypes that were highly resistant to the individual four races used in the current study. However, only two RILs (YY_49 and YY_65) were highly resistant to all four. These two RILs are important genetic resources that can be used by the bean breeding community working on the yellow bean market classes to enhance their ANTH resistance. This would be feasible given the high heritability (0.93) observed for ANTH resistance in this study. The identification of these two RILs is important as they provide an opportunity to circumvent the challenges bean breeders encounter when introgressing resistance from other market classes because of the poor recovery of the yellow seed coat when crossing yellows with other market classes.

Both minor- and major-effect QTLs were identified, suggesting that the observed resistance in the population was controlled by both major and minor QTLs. None of the identified QTLs provided resistance to more than two races, which underscores the race-specific nature of the resistance provided by these QTLs and is consistent with previous reports (Mungalu et al., 2020; Ferreira et al., 2013; Kuwabo et al., 2023; Sinkala et al., 2024). The race-specific resistance of this QTL has partly been attributed to the existence of clusters of R genes at these major-effect loci and individual genes of the plausibly providing resistance to specific races.

The large-effect QTL ANT1.1 identified in the current study provided resistance to races 6 and 81 and overlapped with the well-known *Co-1* locus, which has previously been mapped from the 49,444,405- to 49,828,427-bp genomic region (Paulino et al., 2022). *Co-1* is a multi-allelic Andean locus effective against Andean races and mixed races. Most of the bean grown in Zambia are Andean; therefore, Andean and mixed races of *C. lindemuthianum* are dominant, although Middle American races have also been reported (Sansala et al., 2024). It is interesting to note that ANT1.1 (overlapping with *Co-1*) identified in the current study is in a more productive yellow genetic background that can be used in improving yellow beans instead of getting it from other backgrounds such as from the Andean differentials. Given the multi-allelic nature of the *Co-1*, further studies involving allelism tests may be needed to determine the type of allele associated with ANT1.1. The region where ANT1.1 was identified does contain genes that provide resistance to other diseases such as *Phg1* gene for resistance to angular leaf spot (ALS) (Gonçalves-Vidigal et al., 2011). Therefore, introgressing *Co-1* at ANT1.1 does bring the added benefit of potential resistance to ALS depending on the race.

Three QTLs were identified on Pv10 for resistance to races 6, 7, and 294. Two of these QTLs, ANT10.2 and ANT10.3, were major-effect with an *R*^2^ of 12.2% and 29.3%, respectively. The other QTL was a minor QTL with an *R*^2^ of 6.3%. Previous studies have reported major- and minor-effect QTLs on Pv10. Recently, Lateef et al. (2024) reported a major QTL (provisionally named as Co-18) at the beginning of Pv10 (4.0–4.1 Mbp). This QTL does not co-localize with the three QTLs identified in the current study. Zuiderveen et al. (2016) conducted a GWAS using the Andean panel and reported a QTL for resistance to race 7 on Pv10 (3.8 Mbp). Interestingly, the QTL ANT10.2 (3.8 Mbp) for resistance to race 7 identified in the current study co-localizes with this QTL identified by Zuiderveen et al. (2016). This co-localization suggests that this is a major and stable resistance locus to race 7. The other two identified QTLs in the current study do not overlap with other previously reported QTLs (Lateef et al., 2024). The identification of three QTLs, of which one overlaps with previously identified QTLs, suggests the increasing importance of Pv10 in ANT resistance, particularly in quantitative resistance, which is different from the qualitative resistance provided by the *Co* loci (Lateef et al., 2024). ANT10.2 was located in a genomic “hotspot” for disease resistance genes with the NBC-ARC domain. A total of 14 resistance genes with NB-ARC-LRR and TIR-NBS-LRR domains (**Supplementary** **Table 2**) were identified within the confidence of ANT10.2, and majority of these occurred in an uninterrupted cluster, and these genes potentially play a major role in providing race-specific resistance of this QTL on Pv10. Genes with the NB-ARC domain have previously been reported as candidate genes for ANT resistance QTL (Oblessuc et al., 2015).

A minor QTL, ANT4.1, was identified on Pv04 (with QTL peak at 5.3 Mbp). There are major genes for ANT and ALS resistance reported at the beginning of Pv04. Additionally, the genes for rust (*Ur-5, Ur-14*, and *Ur-Dorado*) and for golden halo blight are also located on Pv04. Downstream of ANT4.1 (5.3 Mbp) identified in the current study is the multi-allelic *Co-3* locus (3.36 Mbp) and *Phg-3* gene for ALS while upstream at 9.08 Mbp is the Andean *Co-15* locus (Sousa et al., 2015), which suggests that this QTL is different from these *Co* genes and Phg-3 for ALS. Despite being a minor-effect QTL, it may be important in providing quantitative resistance and supplementing the qualitative resistance provided by the *Co* loci, and presumably important in slowing down the possible breakdown of the *Co* loci due to race evolution. Five disease resistance genes with the NB-ARC domain (**Supplementary** **Table 2**) were identified as the underlying candidate gene for resistance to race 6 conferred by the QTL ANT4.1.

## Conclusion

Four isolates collected from a major bean-growing region of Zambia were characterized as races 6, 7, 81, and 294, respectively. This is the first report of these races in Zambia. Only two RILs were highly resistant all four races. A total of six QTLs (ANT1.1, ANT3.1, ANT4.1, ANT10.1, ANT10.2, and ANT10.3) were identified as having conferred resistance to the four characterized races. These QTLs explained 6.3%–90.3% of ANT resistance variation among RILs, suggesting that resistance was controlled by both major- and minor-effect loci. A total of 31 disease resistance genes with NB-ARC-LRR and TIR-NBS-LRR domains were identified as candidate genes for ANT1.1 and ANT10.2 at Pv01 and Pv10, respectively. The two RILs with superior resistance to all four races represent a valuable genetic resource to the bean breeding community to improve the yellow beans for ANT resistance while QTL analyses have provided valuable information that can be used to develop a marker-assisted selection strategy for ANT resistance in the yellow bean market class.

## Data Availability

The original contributions presented in the study are included in the article/**Supplementary Material**, further inquiries can be directed to the corresponding author.

## References

[B1] Badiyal A. Dhiman S. Singh A. Rathour R. Pathania A. Katoch S. . (2024). Mapping of adult plant recessive resistance to anthracnose in Indian common bean landrace Baspa/KRC 8. Mol. Biol. Rep. 51, 254. doi: 10.1007/s11033-023-09160-3, PMID: 38302755

[B2] Bassett A. Katuuramu D. N. Song Q. Cichy K. (2021). QTL mapping of seed quality traits including cooking time, flavor, and texture in a yellow dry bean (*Phaseolus vulgaris* L.) population. Front. Plant Sci. 12. doi: 10.3389/fpls.2021.670284, PMID: 34239523 PMC8259628

[B3] Broman K. W. Wu H. Sen Ś. Churchill G. A. (2003). R/qtl: QTL mapping in experimental crosses. Bioinformatics 19, 889–890. doi: 10.1093/bioinformatics/btg112, PMID: 12724300

[B4] Broughton W. J. Hernández G. Blair M. Beebe S. Gepts P. Vanderleyden J. (2003). Beans (Phaseolus spp.)–model food legumes. Plant Soil 252, 55–128. doi: 10.1023/A:1024146710611, PMID: 40797221

[B5] Buruchara R. Chirwa R. Sperling I. Mukankusi C. Muthoni R. Abang M. M. (2011). Development and derivery of bean varieties in Africa: The Pan-African Bean Researc Alliance (PABRA) model. Afr. Crop Sci. J. 19, 227–245.

[B6] Cichy K. Wiesinger J. Mendoza F. (2015). Genetic diversity and genome-wide association analysis of cooking time in dry bean (Phaseolus vulgaris L.). Theor. Appl. Genet. 128, 1555–1567. doi: 10.1007/s00122-015-2531-z, PMID: 26003191

[B7] Ferreira J. J. Campa A. Kelly J. D. (2013). “Organization of genes conferring resistance to anthracnose in common bean,” In Translational Genomics for Crop Breeding, Volume I: Biotic stresses, e.d. R.K. Varshney and R (Tuberosa (Chichester, UK: John Wiley and Sons), 151–181.

[B8] Geffroy V. Sicard D. de Oliveira J. C. Sévignac M. Cohen S. Gepts P. . (1999). Identification of an ancestral resistance gene cluster involved in the co-evolution process between Phaseolus vulgaris and its fungal pathogen Colletotrichum lindemuthianum. Mol. Plant-Microbe Interact. 12, 774–784. doi: 10.1094/MPMI.1999.12.9.774, PMID: 10494630

[B9] Gonçalves-Vidigal M. C. Cruz A. S. Garcia A. Kami J. Vidigal Filho P. S. Sousa L. L. . (2011). Linkage mapping of the Phg-1 and Co-1–4 genes for resistance to angular leaf spot and anthracnose in the common bean cultivar AND 277. Theor. Appl. Genet. 122, 893–903. doi: 10.1007/s00122-010-1496-1, PMID: 21113774 PMC3043234

[B10] Kachapulula J. S. Kuwabo K. Hamabwe S. M. Nkandela M. Mukuma C. Soler-Garzón A. . (2025). Quantitative trait loci analysis for anthracnose resistance in a population derived from andean varieties bukoba and kijivu of common bean (*Phaseolus vulgaris* L.). Plant Breed. 144, 432–439. doi: 10.1111/pbr.13264, PMID: 41875165

[B11] Kachapulula P. (2010). Characterisation of Southern African *Colletotrichum lindemuthianum* isolates using neutral and selective genetic markers. [Master’s thesis] ([Kampala, Uganda]: Makerere University).

[B12] Kuwabo K. Hamabwe S. M. Kachapulula P. Cichy K. Parker T. Mukuma C. . (2023). Genome-wide association analysis of anthracnose resistance in the Yellow Bean Collection of Common Bean. PloS One 18, e0293291. doi: 10.1371/journal.pone.0293291, PMID: 37948396 PMC10637669

[B13] Lateef I. Katoch S. Katoch A. Badiyal A. Pathania A. Dhiman S. . (2024). Fine mapping of a new common bean anthracnose resistance gene (Co-18) to the proximal end of Pv10 in Indian landrace KRC-5. Theor. Appl. Genet. 137, 32. doi: 10.1007/s00122-023-04539-z, PMID: 38270625

[B14] Lorieux M. (2012). MapDisto: fast and efficient computation of genetic linkage maps. Mol. Breed. 30, 1231–1235. doi: 10.1007/s11032-012-9706-y, PMID: 41878318

[B15] Mahuku G. S. Riascos J. J. (2004). Virulence and molecular diversity within *Colletotrichum lindemuthianum* isolates from Andean and Mesoamerican bean varieties and regions. Eur. J. Plant Pathol. 110, 253–263. doi: 10.1023/B:EJPP.0000019795.18984.74, PMID: 38124636

[B16] Mohammed A. (2013). An overview of distribution, biology and the management of common bean anthracnose. J. Plant Pathol. Microbiol. 4, 1–6. doi: 10.4172/2157-7471.1000193, PMID: 39887974

[B17] Mungalu H. Sansala M. Hamabwe S. Mukuma C. Gepts P. Kelly J. D. . (2020). Identification of race-specific quantitative trait loci for resistance to *Colletotrichum lindemuthianum* in an Andean population of common bean. Crop Sci. 60, 2843–2856. doi: 10.1002/csc2.20191, PMID: 41881430

[B18] Mwense B. P. Hamabwe S. M. Kuwabo K. Mataa M. Miklas P. N. Mukuma C. . (2024). Evaluation of pinto genotypes of common bean for resistance to anthracnose. Legume Sci. 6. doi: 10.1002/leg3.228, PMID: 41881430

[B19] Nalupya Z. Hamabwe S. Mukuma C. Lungu D. Gepts P. Kamfwa K. (2021). Characterization of *Colletotrichum lindemuthianum* races in Zambia and evaluation of the CIAT Phaseolus core collection for resistance to anthracnose. Plant Dis. 105, 3939–3945. doi: 10.1094/PDIS-02-21-0363-RE, PMID: 33988467

[B20] Nunes M. P. B. A. Gonçalves-Vidigal M. C. Martins V. S. R. Xavier L. F. S. Valentini G. Vaz Bisneta M. . (2021). Relationship of *Colletotrichum lindemuthianum* races and resistance loci in the Phaseolus vulgaris L. genome. Crop Sci. 61, 3877–3893. doi: 10.1002/csc2.20601, PMID: 41881430

[B21] Oblessuc P. R. Francisco C. Melotto M. (2015). The Co-4 locus on chromosome Pv08 contains a unique cluster of 18 COK-4 genes and is regulated by immune response in common bean. Theor. Appl. Genet. 128, 1193–1208. doi: 10.1007/s00122-015-2500-6, PMID: 25805316

[B22] Pastor-Corrales M. A. (1991). Estandarización de variedades diferenciales y designación de razas de *Colletotrichum lindemuthianum*. Phytopathology 81, 694.

[B23] Paulino P. P. S. Gonçalves-Vidigal M. C. Bisneta M. V. Vidigal Filho P. S. Nunes M. P. B. A. Xavier L. F. S. . (2022). Occurrence of anthracnose pathogen races and resistance genes in common bean across 30 years in Brazil. Agron. Sci. Biotechnol. 8, 1–21. doi: 10.33158/ASB.r140.v8.2022

[B24] Rodríguez-Suárez C. Ferreira J. J. Campa A. Pañeda A. Giraldez R. (2008). Molecular mapping and intra-cluster recombination between anthracnose race-specific resistance genes in the common bean differential cultivars Mexico 222 and Widusa. Theor. Appl. Genet. 116, 807–814. doi: 10.1007/s00122-008-0714-6, PMID: 18210079

[B25] Sadohara R. Long Y. Izquierdo P. Urrea C. A. Morris D. Cichy K. (2022). Seed coat color genetics and genotype× environment effects in yellow beans via machine-learning and genome-wide association. Plant Genome 15, e20173. doi: 10.1002/tpg2.20173, PMID: 34817119 PMC12806898

[B26] Sansala M. Kuwabo K. Hamabwe S. M. Kachapulula P. Parker T. Mukuma C. . (2024). Race structure and molecular diversity of *Colletotrichum lindemuthianum* of common bean in Zambia. Plant Dis. 108, 857–865. doi: 10.1094/PDIS-01-23-0143-RE, PMID: 37622270

[B27] Sichilima T. Mapemba L. Tembo G. (2016). Drivers of dry common beans trade in Lusaka, Zambia: a trader’s perspective. Sustain. Agric. Res. 5, 15–26. doi: 10.5539/sar.v5n2p15

[B28] Singh S. P. Schwartz H. F. (2010). Breeding common bean for resistance to diseases: A review. Crop Sci. 50, 2199–2223. doi: 10.2135/cropsci2009.03.0163

[B29] Sinkala W. Hamabwe S. Kuwabo K. Mukuma C. Kamfwa K. (2024). Genome-wide association analysis of resistance to anthracnose in the Middle American Diversity Panel of common bean (Phaseolus vulgaris L.). Crop Sci. 64, 3126–3134. doi: 10.1002/csc2.21335, PMID: 41881430

[B30] Sousa L. L. Gonçalves A. O. Gonçalves-Vidigal M. C. Lacanallo G. F. Fernandez A. C. Awale H. . (2015). Genetic characterization and mapping of anthracnose resistance of common bean landrace cultivar Corinthiano. Crop Sci. 55, 1900–1910. doi: 10.2135/cropsci2014.09.0604

[B31] Uebersax M. A. Cichy K. A. Gomez F. E. Porch T. G. Heitholt J. Osorno J. M. . (2022). Dry beans (Phaseolus vulgaris L.) as a vital component of sustainable agriculture and food security—A review. Legume Sci. 5. doi: 10.1002/leg3.155, PMID: 41881430

[B32] van Schoohoven A. Pastor-Corrales M. A. (1987). Standard system for the evaluation of bean germplasm (Cali, Colombia: CIAT).

[B33] Voorrips R. E. (2002). Mapchart: Software for the graphical presentation of linkage maps and QTLs. J. Hered. 93, 77–78. doi: 10.1093/jhered/93.1.77, PMID: 12011185

[B34] Wiesinger J. A. Cichy K. A. Glahn R. P. Grusak M. A. Brick M. A. Thompson H. J. . (2016). Demonstrating a nutritional advantage to the fast-cooking dry bean (Phaseolus vulgaris L.). J. Agric. Food Chem. 64, 8592–8603. doi: 10.1021/acs.jafc.6b03100, PMID: 27754657

[B35] Wiesinger J. Cichy K. Tako E. Glahn R. (2018). The fast cooking and enhanced iron bioavailability properties of the Manteca yellow bean (*Phaseolus vulgaris* L.). Nutrients 10, 1609. doi: 10.3390/nu10111609, PMID: 30388772 PMC6266362

[B36] Young R. A. Kelly J. D. (1996). Characterization of the genetic resistance to *Colletotrichum lindemuthianum* in common bean differential cultivars. Plant Dis 80, 650–654. doi: 10.1094/PD-80-0650, PMID: 40211709

[B37] Young R. A. Melotto M. Nodari R. O. Kelly J. D. (1998). Marker-assisted dissection of the oligogenic anthracnose resistance in the common bean cultivar, ‘G2333’. Theor. Appl. Genet. 96, 87–94. doi: 10.1007/s001220050713, PMID: 41878318

[B38] Zuiderveen G. H. Padder B. A. Kamfwa K. Song Q. Kelly J. D. (2016). Genome-wide association study of anthracnose resistance in Andean beans (*Phaseolus vulgaris*). PloS One 11. doi: 10.1371/journal.pone.0156391, PMID: 27270627 PMC4894742

[B39] Zulu M. (2005). Race identification and distribution of bean anthracnose (*Colletotrichum lindemuthianum*) in major bean growing areas of Zambia. [Master’s thesis] ([Lusaka, Zambia]: University of Zambia).

