# Correction: Mapping of quantitative trait locus for resistance to anthracnose in a population derived from genotypes PI 527538 and Ervilha of common bean

**DOI:** 10.3389/fpls.2026.1767038

**Published:** 2026-03-26

**Authors:** Mwiinga Mulube, Swivia Hamabwe, Kuwabo Kuwabo, Modreen Chinji, Mukuni Nkandela, Joseph Botha, Brian Mwense, Langa Tembo, Davies Lungu, Chikoti Mukuma, Travis Parker, Kelvin Kamfwa

**Affiliations:** 1 Department of Plant Sciences, University of Zambia, Lusaka, Zambia; 2 Zambia Agricultural Research Institute, Kasama, Zambia; 3 Department of Plant Sciences, University of California, Davis, Davis, CA, United States

**Keywords:** *Colletotrichum lindemuthianum*, quantitative trait locus, candidate genes, recombinant inbred lines, race

There was a mistake in **Figure 1** and **2** as published. The figure images were swapped, but the captions were correct. That is, the linkage map figure originally presented as **Figure 1** should be **Figure 2**, while the frequency table distribution graph presented as **Figure 2** should be **Figure 1**.

**Figure 1:**
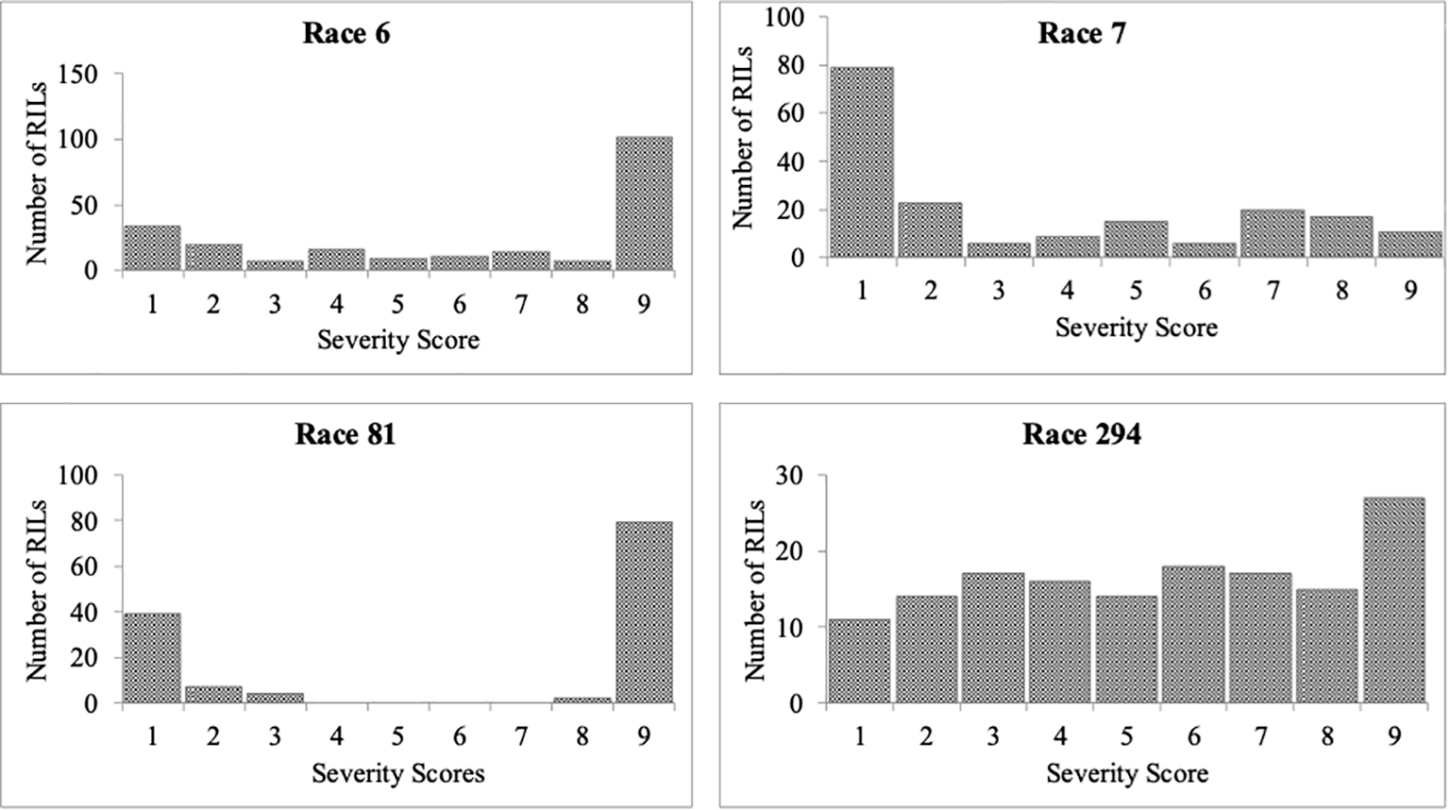
Frequency distributions of severity scores for *Colletotrichum lindemuthianum* races 6, 7, 81, and 294 inoculated on recombinant inbred lines derived from a cross of PI 527538 and Ervilha. This should be the bare graph (also known as histogram).

**Figure 2:**
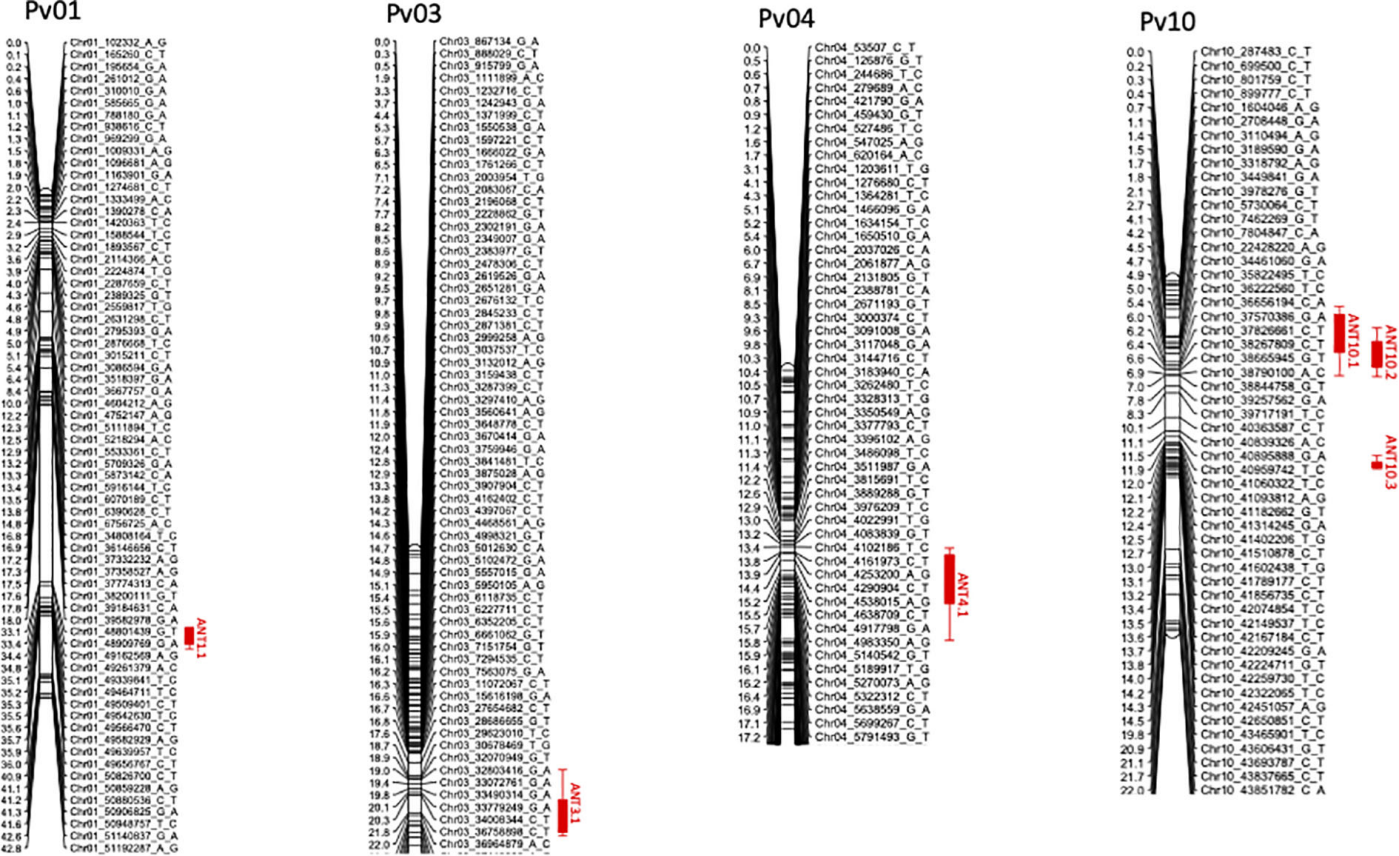
Linkage maps for chromosomes Pv01, Pv03, Pv04, and Pv10 with positions for QTL ANT1.1 (for races 6 and 81), ANT3.1 (race 294), ANT4.1 (race 6), ANT10.1 (race 6), ANT10.2 (race 7), and ANT10.3 (race 294). This should for the linkage maps.

The corrected **Figure 1** and **Figure 2** appear below.

The original version of this article has been updated.

